# Outcomes and Complications of 33 Soft-Milled Cobalt-Chromium-Ceramic Full-Arch Screw-Retained Implant-Supported Prostheses: A Retrospective Study with up to 10-Year Follow-Up

**DOI:** 10.3390/jfb14030157

**Published:** 2023-03-16

**Authors:** Hadas Heller, Ilan Beitlitum, Tomer Goldberger, Alona Emodi-Perlman, Shifra Levartovsky

**Affiliations:** 1 Department of Oral Rehabilitation, The Maurice and Gabriela Goldschleger School of Dental Medicine, Tel Aviv University, Tel Aviv 6997801, Israel; 2 Department of Periodontology and Dental Implantology, The Maurice and Gabriela Goldschleger School of Dental Medicine, Tel Aviv University, Tel Aviv 6997801, Israel; 3 Department of Endodontics, The Maurice and Gabriela Goldschleger School of Dental Medicine, Tel Aviv University, Tel Aviv 6997801, Israel

**Keywords:** cobalt-chromium, soft-mill, full arch, porcelain, implant

## Abstract

This retrospective study assessed outcomes and complications related to implants and prostheses in edentulous patients treated with soft-milled cobalt-chromium-ceramic full-arch screw-retained implant-supported prostheses (SCCSIPs). After the final prosthesis was delivered, patients participated in an annual dental check-up program, including clinical and radiographic assessments. Outcomes of implants and prostheses were evaluated, and biological and technical complications were categorized as major or minor. Implant and prosthesis cumulative survival rates were assessed using the life table analysis. A total of twenty-five participants (mean age 63.6 ± 7.3 years) with 33 SCCSIPs were observed for a mean of 68.9 ± 27.9 months (range 1–10 years). A total of 7 out of 245 implants were lost, with no effect on prosthesis survival, leading to cumulative survival rates of 97.1% for implants and 100% for prostheses. The most recurrent minor and major biological complications were soft tissue recession (9%) and late implant failure (2.8%). Among 25 technical complications, porcelain fracture was the only major technical complication, requiring prosthesis removal (1%). Porcelain chipping was the most frequent minor technical complication, affecting 21 crowns (5.4%), which required polishing only. At the end of the follow-up, 69.7% of the prostheses were free of technical complications. Within the limitations of this study, SCCSIP showed promising clinical performance after 1–10 years.

## 1. Introduction

Full-arch, implant-retained prostheses have been proven a successful treatment for patients with completely edentulous jaws. The available restorative materials for these prostheses are metal-ceramic, metal-acrylic resin, veneered zirconia, and monolithic zirconia. Veneered or monolithic zirconia is usually favored due to its esthetic outcomes and biocompatibility. However, a retrospective study that evaluated prosthetic complications in complete-arch prostheses of monolithic or micro-veneered zirconia, with a mean follow-up of 72.35 months, found a very high probability of complications (50%) [1]. On the other hand, a recent systematic review that compared the biological and technical outcomes of metal-resin vs. metal-ceramic full arch implant-supported prostheses reported that a significantly greater incidence of developing biologic complications of peri-implantitis, mucositis, and mucosal recession around the implants, as well as technical complication, such as wear and veneer fracture, were in the metal-resin group [2]. In another retrospective study, significantly more soft tissue hypertrophy and plaque accumulation were found around implants supporting metal-acrylic resin prostheses compared to metal-ceramic [3]. Therefore, although metal-ceramic has disadvantages, it was considered the first choice for implant-supported fixed-partial dentures in a consensus study on prosthodontics and implant dentistry [4]. It is important to note that a full arch implant-supported prosthesis is less prone to technical complications than a single implant-supported crown. A probable explanation might be that in a full arch, the torsion forces are supported by multiple implants [5,6].

Using computer-aided design and computer-aided manufacturing (CAD-CAM) technology, cobalt-chromium alloys (Co-Cr) became extremely popular for metal-ceramic prostheses, with two CAM technologies: additive manufacturing and subtractive manufacturing (milling) [7,8]. Both methods were considered to have adequate adhesion strength to porcelain, but because the additive manufacturing technique requires very expensive technical equipment, the milling technique, also known as milling/post-sintering (ML/PS), became more popular in dental laboratories using available CAD/CAM systems. The principal advantage of this technique is less stress on the milling tools because ‘soft’ blanks are milled before the sintering step [7,8]. Revilla-León et al. compared the implant abutment-prosthesis interface before and after ceramic veneering of frameworks fabricated using milling (subtractive) or selective laser melting (additive) technologies and found no significant differences between the groups, as both had clinically acceptable fits [9]. Kim et al. examined the clinical accuracy of fixed partial prostheses fabricated by the milling/post-sintering method using a pre-sintered Co-Cr alloy. They found that its overall marginal fit was comparable to the prostheses manufactured using conventional casting [10].

According to these data, the use of full arch soft-milled Co-Cr screw-retained implant-supported prostheses should be examined. A recent in vitro study compared the accuracy of Co-Cr complete arch screw-retained implant-supported fixed dental prosthesis fabricated with conventional casting, milling, or additive manufacturing technology. It was demonstrated that all three fabrication techniques exhibited acceptable, marginal fits; however, milling resulted in the most accurate marginal fit [11]. An in vivo study that retrospectively assessed the outcomes and complications of soft-milled Co-Cr-ceramic complete arch screw-retained implant-supported prostheses over a mean clinical follow-up of 4 years and found high implant and prosthesis survival rates, while 33% of the prostheses had technical complications [12]. 

As far as we know, studies reporting the long-term outcomes of complete arch screw-retained implant-supported metal-ceramic prostheses using soft-milled Co-Cr technology are scarce. Therefore, the aim of the current study was to retrospectively assess implant and prosthesis outcomes and complications among edentulous patients treated with soft-milled Co-Cr ceramic full-arch screw-retained implant-supported prostheses (SCCSIPs), after a follow-up period of 1–10 years. 

## 2. Materials and Methods

### 2.1. Study Sample

The electronic documents of all patients who had received soft-milled cobalt-chromium-ceramic full-arch screw-retained implant-supported prostheses through the graduate program of prosthodontics in the School of Dental Medicine or the private practice of the program director (SL) were reviewed according to the following inclusion and exclusion criteria. 

Patients with at least one edentulous jaw that had been rehabilitated, with preoperative, intraoperative, and postoperative clinical and radiographic data, and with a minimum follow-up of one year after the delivery of the final prosthesis, were included. Exclusion criteria were: patients diagnosed with bruxism, uncontrolled medical condition, aggressive periodontal condition, SCCSIP in at least one edentulous jaw, incomplete records, and unavailable for a recall less than one year after inserting the definitive prosthesis.

All patients were registered in a maintenance care program after the final prosthesis insertion and were monitored with radiographic and clinical examination annually, except for the first recall appointment, which was six months after the final prosthesis delivery. In addition, these patients were called to attend a recall appointment and were informed about the current study objectives. Those who agreed to participate provided written informed consent. 

The detailed study protocol was previously described [12]. All patients received the same surgical and prosthetic protocol. Clinical examination, panoramic X-rays, and computed tomography scans were performed. The treatment plan was established after evaluating the functional and esthetic needs of each individual. Two implant systems (Tapered Screw-Vent^®^, Zimmer Biomet, Palm Beach Gardens, FL, USA, and Lance Implant, MIS/Divident, Or-Yehuda, Israel) were used for the surgical phase. Each edentulous jaw received 4–10 implants.

After achieving osseointegration, multi-unit abutments were connected with an insertion torque of 30 Ncm, according to the manufacturer’s recommendations. According to the vertical occlusal dimension, the provisional prostheses were constructed and placed [13,14]. Functional, esthetic, and phonetic criteria were evaluated for at least two months. No signs or symptoms of discomfort or bruxism were noticed. 

**Figure 1:**
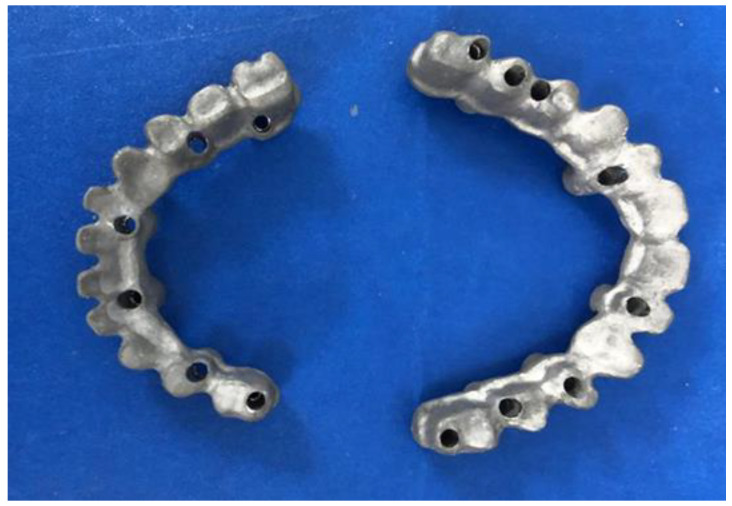
Double arch milling/post-sintering Co-Cr frameworks.

**Figure 2:**
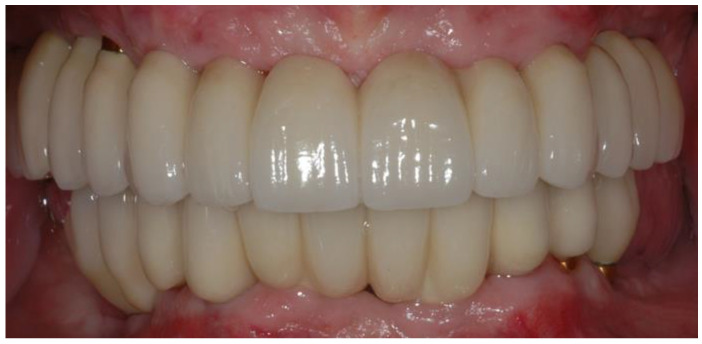
Final prostheses (SCCSIPs) after delivery.

Within six to nine months after implant placement, final impressions were performed. The final prostheses were fabricated with milling/post-sintering Co-Cr full-arch screw-retained implant-supported metal frames (Ceramill Sintron, Amann Girrbach AG, Koblach, Austria or Zirkonzahn, Gais, Italy) (Figure 1) and were evaluated for accuracy and passivity using radiographs and the 1-screw test [15]. Thereafter, the metal frames were veneered with feldspathic porcelain (Super Porcelain EX-3, Kuraray Noritake Dental Inc., Tokyo, Japan) (Figure 2). There were no cantilevers, and the opposing arches were either natural teeth or full arch implant-supported prostheses, as seen in the patient presented in Figure 2. 

All the functional and esthetic parameters were verified with the definitive prostheses (SCCSIPs). The occlusal scheme was adjusted with articulating paper to achieve maximum intercuspation with mutual protection. Finally, all prostheses were tightened to the abutments with an insertion torque of 25 Ncm, according to the manufacturer’s recommendations, and the screw access openings were filled with a composite resin (G-ænial™ Universal Injectable, GC, Tokyo, Japan).

### 2.2. Data Collection at the Recall Appointments

At each recall appointment, clinical and radiographic assessments were conducted to evaluate the survival and success of all implants and prostheses. The periapical radiographs, performed using the long cone technique, were compared to the radiographs taken at the time of the final prosthesis delivery, and two examiners (H.H. and S.L.) assessed all records. 

Peri-implant parameters, including probing depths and bleeding index, were assessed for each implant. Minor biological complications were considered as peri-implant mucositis and the presence of soft tissue recession. Peri-implant mucositis was diagnosed based on bleeding on probing and/or the presence of peri-implant suppuration without radiographic loss of supporting bone around a functioning implant [16]. Major biological complications were defined as a diagnosis of peri-implantitis or implant failure. Peri-implantitis was assessed, according to the Consensus report of the 2017 World Workshop on periodontal and peri-implant diseases and conditions, as bleeding and/or suppuration on gentle probing, probing depths of ≥6 mm, and bone levels ≥3 mm apical to the most coronal portion of the intraosseous part of the implant [17]. Implant failure was determined when an implant did not survive during the follow-up period [18].

Prosthetic complications were also divided into minor and major according to previously reported criteria [19,20]. Specifically, minor complications were those that could be treated chairside without the need to remove the prosthesis, such as minor chipping of porcelain and loosening of an occlusal screw. Major complications required the removal of the prosthesis because of an implant fracture, an abutment, a screw, a framework, or prosthetic material.

### 2.3. Statistical Methods and Synthesis of Results

Descriptive statistics of patient age and follow-up duration were analyzed using SPSS, version 27.0 (IBM Corp., Armonk, NY, USA). Cumulative survival rates of the implants and the SCCSIP prostheses were assessed by Life table analysis. Power analysis was performed. A sample size of n = 37 provided 80% power at a confidence of 95% to detect a failure rate of 5%.

## 3. Results

The initial pool of patients was 16 females and 12 males who complied with the inclusion criteria. Three did not attend the recall appointment, two did not respond to the invitation to participate in the study, and one was deceased. Therefore, 25 patients (15 females, 10 males) were included in the study. The participants ranged from 47 to 74 years of age (mean: 63.6 ± 7.3 years) and were followed for a mean observation period of 68.9 ± 27.9 months (range 1–10 years). They were treated with 245 implants, supporting 33 SCCSIPs (19 in the maxilla and 14 in the mandible), with a total of 387 crown units. Descriptive statistics are reported in Table 1.

The current study provides a large sample size at the implant (245)/crown unit (387) level, but at the prosthesis level (33), a small sample size was used. 

A total of 7 of the 245 implants failed during the follow-up period, leading to a 97.1% cumulative implant survival rate at 68.9 ± 27.9 months. Four implants (Tapered Screw-Vent^®^, Zimmer Biomet, Palm Beach Gardens, FL, USA) failed in two patients, and the other three implants (Lance Implant, MIS/Divident, Or Yehuda, Israel) failed in three other patients. None of these patients were smokers or had aggressive periodontitis. The life table analysis of the implant survival rate is shown in Table 2.

The failed implants were removed without affecting the prostheses’ survival because of the number of supporting implants in each prosthesis. After removing the failed implants, the screw holes in the prostheses were completed with composite resin, and the SCCSIPs were rescrewed on the abutments. Therefore, the cumulative prosthesis survival rate remained 100%. Table 3 shows the life table analysis concerning SCCSIP survival. 

Descriptive statistics for major and minor biological and technical complications are presented in Table 4.

At the implant level, 37 minor biological complications and 12 major complications were recorded in 11 SCCSIPs. The minor biological complications were soft tissue recession and peri-implant mucositis; peri-implantitis and implant failure were the major complications. The most recurrent minor biologic complication was soft tissue recession (9%), followed by peri-implant mucositis (6.1%). Late implant failure was the most frequently noticed major biological complication (2.8%), followed by peri-implantitis (2%).

**Figure 3:**
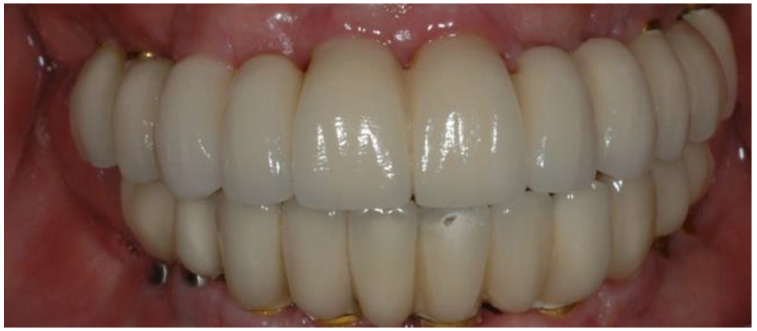
Porcelain fracture in the lower left central incisor, 3 months post-delivery. Repair required prosthesis removal.

Although the overall SCCSIP survival rate was 100%, 10 of the 33 prostheses (30.3%) were affected by technical complications. Of the 387 crown units, 25 technical complications (6.4%) were noticed. The most recurrent minor technical complication was slight porcelain chipping, influencing 21 crowns (5.4%), which required polishing only. Porcelain fracture that required prosthesis removal was the only major technical complication (1%) and affected 4 lower prostheses in 4 patients (Figure 3).

All porcelain fractures occurred within the first six months after definitive prosthesis insertion. The prostheses were removed, and the veneered feldspathic porcelain was sent to the lab for repair. The SCCSIPs were reinserted without further complications.

At the end of the observation period, 69.7% of the prostheses were free of technical complications.

## 4. Discussion

The current study assessed the outcomes and complications of prostheses and implants in edentulous patients treated with soft-milled cobalt-chromium-ceramic full-arch screw-retained implant-supported prostheses, with a follow-up period of 1–10 years. As noted, fabrication of Co-Cr dental restorations through soft milling overcomes the main disadvantage of handling the rigid ‘solid’ blank, which increases the tool and machine wear. Another disadvantage of milling the ‘solid’ Co-Cr blanks is the high cost of acquisition and maintenance. In this process of milling the ‘soft’ Co-Cr blanks, the Co-Cr powder is distributed in a binder material capable of burn-out. Then, the milled prosthesis is sintered, subsequently, to its full density in a high-temperature sintering furnace under an argon protective gas atmosphere at approximately 1300 °C [21]. A study that compared the mechanical characteristic of Co-Cr alloys produced by three CAD/CAM-based processing techniques to those produced by the casting technique showed superior properties of the selective laser melting and milling/post-sintering techniques [21]. Another study, that compared the mechanical properties of Co-Cr alloys manufactured by three CAD-CAM techniques with the casting process, found considerably greater elongation of the milling/post-sintering Co-Cr compared to the other techniques [22]. These studies reported that the milling/post-sintering technique might hold promise in fabricating Co-Cr dental restorations [21,22]. Yet, to our knowledge, only one study reported follow-up using milling/post-sintering Co-Cr frameworks for full-arch implant-supported metal-ceramic prostheses [12].

The results of the current study include a 100% cumulative survival rate for the prostheses and 97.1% for the implants (due to seven implant failures) after a mean observation period of 68.9 ± 27.9 months (range 1–10 years). Some of our findings are consistent with previous reports concerning the survival and success of metal-ceramic implant-supported fixed complete-arch prostheses. Chochlidakis et al. assessed survival rates and prosthetic complications of implant-fixed complete dental prostheses with up to 5 years of follow-up [23]. A total of 48 prostheses were included; 38 were metal-acrylic resin, and 10 were metal-ceramic. Among the 48 prostheses, five failed during the follow-up period, showing a cumulative prosthesis survival rate of 88%. It should be noted that all failures occurred in metal-acrylic resin prostheses. On the other hand, the survival rate of the metal-ceramic prostheses was 100%. In another retrospective study that assessed the implant survival and biological complications of 43 implant-supported fixed complete dental prostheses with up to 5 years of follow-up, none of the implants failed [3]. Gonzalez et al. evaluated the biological and mechanical-technical complications, and survival rate of implants of complete-arch implant-supported metal-ceramic prostheses, during 5 years of follow-up [24]. The cumulative survival rate of the implants was 99.8%, and 98.8% for the prostheses. In a retrospective study with 1 to 12 years of follow-up, Papaspyridakos et al. found cumulative implant and metal-ceramic prosthesis survival rates of 99.4% and 98.2%, respectively. Overall, based on the current and aforementioned studies, the survival rates of restorations and implants in complete-arch implant-supported metal-ceramic prostheses seem to be very high [25]. 

Although a systematic review of implant-supported fixed full-arch dentures found that the type of the prosthetic material had no clinically relevant influence on implant and prosthetic survival rates [26], another recent systematic review reported a higher incidence of biological and prosthetic complications, including greater risk of peri-implantitis, with metal-acrylic resin compared to metal-ceramic implant-supported fixed complete dental prostheses [2]. In the current study, peri-implant mucositis was the most recurrent minor biological complication (6.1%), while late implant failure was the most frequently-observed major biological complication (2.8%), followed by peri-implantitis (2%). These results are inconsistent with those of Chochlidakis et al., who found peri-implant mucositis in 53% of the implant sites. Peri-implantitis, the most common major biological complication, affected 4.0% of the implants, but without any implant failure, in an up to 5-year retrospective study [3]. A probable explanation for the differences in prevalence rates might be because our retrospective study had a longer follow-up period (up to 10 years), in which some of the implants with peri-implantitis eventually failed. On the other hand, our results agree with those of another retrospective study of 5–12 Year Follow-up. At the end of the follow-up period (12 years), the cumulative rate of peri-implantitis at the implant level was 4.4% in the complete-arch screw-retained group and 2.2% in the complete-arch telescopic-retained group with no significant difference between them [27]. In the aforementioned study, two implant systems were used, the Branemark and the Ankylos systems, while we used other implant systems, the Zimmer and the MIS systems.

Regarding technical complications, we found that porcelain chipping was the most recurrent minor technical complication (5.4% of the 387 crown units), which required only polishing. This result is comparable with that of a previous retrospective study that also assessed the success of SCCSIPs, but for a shorter follow-up period [19]. Papaspyridakos et al. assessed outcomes and complications of 55 metal-ceramic implant-supported fixed full-arch prostheses and reported higher estimated porcelain chipping rates on the crown unit level: 13.0% after 5 years and 26% after 10 years [28]. However, at the prosthesis level, we found that the complication rate due to minor porcelain chipping was 27.3%, which agrees with the result of Chochlidakis et al., who found a complication rate of 26.67% at the prosthesis level due to minor chipping of veneering material [23]. 

A major porcelain fracture that required prostheses removal was found in 4 lower prostheses in 4 patients, for a complication rate of 12.1% at the prosthesis level. Chochlidakis et al. reported a complication rate of 31.67% at the prosthesis level due to major chipping of veneering material [23]. This high failure rate was probably because most of their prostheses were metal-acrylic resin. Nikellis et al. also reported a total complication rate of 39.29% at the prosthesis level due to major chipping of acrylic veneering material [29].

No cases of abutment, screw, or framework fractures were observed in the current study. This result is comparable to previous retrospective studies of full-arch implant-supported prostheses [12,28,30]. This is probably due to the concept of cross-arch stabilization in which the occlusal forces are distributed evenly among the implants and the abutments in the prosthesis. In those studies where abutment, screw, or framework fractures did occur, the percentage of these technical complications was very small compared to other complications, such as major wear of prosthetic material or minor porcelain chipping [23,29].

Overall, at the end of the observation period, our estimated cumulative rate for “prostheses free of technical complications” was 69.7%. Papaspyridakos et al. reported estimated cumulative rates of 56.4% at 5 years and 9.8% at 10 years for “prostheses free of technical complications” after a mean clinical follow-up of 5 years [28], while another study found a cumulative rate of 80.3% at 5 years, with 45.4% of “prostheses free of major technical complications” after a mean follow-up period of 5.1 years [30]. 

An interesting result was the four major porcelain fractures seen in our study. These occurred only in the anterior lower arch of four patients during the six months following definitive prosthesis insertion. This is maybe due to deformity of the mandible, assumed to be mandibular flexure, which occurs during jaw movements. After the prostheses were removed and the veneered feldspathic porcelains were repaired in the lab, the SCCSIPs were reinserted with no further complications. A recent review concluded that mandibular flexure might be more pronounced in periodontal patients and implant-supported restorations, compared to natural teeth, due to differences in the forces absorbed by the periodontal apparatus. The authors suggested that for implant-retained restorations, it is advisable to split the prosthesis into two or three components [31]. This may prevent the major porcelain fracture immediately after prosthesis insertion. On the other hand, it may increase the other technical complications that may occur later, such as abutment, screw, or framework fractures.

A limitation of the current study was that, due to its retrospective design, clinical procedures were not standardized, and data were based on subjective documentation of procedures and appointments. Other limitations were the small sample size of the prostheses and the short follow-up period. In addition, both implant systems had the same connection–internal hex and the same screw material—titanium. The lack of a control group can also be considered a limitation. Additional, long-term, randomized, controlled studies with larger sample sizes are needed to evaluate the clinical performance of these complete-arch restorations to assist clinicians in choosing the correct framework for implant rehabilitation of edentulous patients.

## 5. Conclusions

Within the limitations of this retrospective study, we may assume that soft-milled cobalt-chromium-ceramic full-arch screw-retained implant-supported prostheses in edentulous patients have high cumulative survival rates of 97.1% for implants and 100% for prostheses. The most frequent minor and major biological complications were soft tissue recession (9%) and late implant failure (2.8%), respectively. Among 25 technical complications, porcelain fracture was the only major technical complication, requiring prosthesis removal (1%), and porcelain chipping was the most recurrent minor technical complication (5.4%), which required polishing only. At the end of the follow-up, 69.7% of the prostheses were free of technical complications.

## Figures and Tables

**Table 1:** Descriptive overview of participants, location of SCCSIPs, and number of implants and crown units.

Characteristics	Number
Participants	25
Mean age Female/male	63.6 years 15/10
Number of prostheses	33
Maxilla/mandible	19/14
Number of implants	245
Number of crown units Mean time follow-up	387 68.9 months

**Table 2:** Life table analysis of implant survival.

Interval (Years)	No. of Implants	No. of Failures	Interval Survival Rate (%)	Cumulative Survival Rate (%)
0–1	245	0	100	100
1–2	226	1	99.5	99.6
2–3	196	0	100	99.6
3–4	196	0	100	99.6
4–5	140	0	100	99.6
5–6	105	1	99.0	99.2
6–7	80	2	97.5	98.4
7–8	56	2	96.4	97.5
8–9	37	0	100	97.5
9–10	23	1	95.6	97.1

**Table 3:** Life table analysis of SCCSIP survival.

Interval (Years)	No. of SCCSIP	No. of Crown Units	No. of Failures	Interval Survival Rate (%)	Cumulative Survival Rate (%)
0–1	33	387	0	100	100
1–2	31	363	0	100	100
2–3	28	328	0	100	100
3–4	28	328	0	100	100
4–5	20	248	0	100	100
5–6	15	190	0	100	100
6–7	11	143	0	100	100
7–8	7	85	0	100	100
8–9	5	57	0	100	100
9–10	3	35	0	100	100

**Table 4:** Distribution of biological and technical complications.

Complication	No. of Implants Affected	No. of Prostheses Affected	Percentage of Complications	
**Biological**			**Implants (%)**	**Prostheses (%)**
**Minor complications**				
Soft tissue recession	22	10	22/246 (9.0)	10/30 (30.3)
Peri-implant mucositis	15	7	15/245 (6.1)	7/33 (21.2)
**Major complications**				
Peri-implantitis	5	4	5/245 (2.0)	4/33 (12.1)
Implant failure	7	5	7/245 (2.8)	5/33 (15.1)
Total	49	11	49/245 (20.0)	11/33 (33.3)
**Technical**			**Crown (%)**	**Prosthesis (%)**
**Minor complications**				
Porcelain chipping	21	9	21/387 (5.4)	9/33 (27.3)
Loosening of a screw	0	0	0	
**Major complications**				
Porcelain fracture	4	4	4/387 (1)	4/33 (12.1)
Abutment fracture	0	0	0	
Fracture of a screw	0	0	0	
Fracture of framework	0	0	0	
Total	25	10	25/387 (6.4)	10/33 (30.3)

## Data Availability

The data that support the findings of this study are available on request from the corresponding author. The data are not publicly available due to privacy and ethical restrictions.
